# [η^5^-(Phenyl­ethyn­yl)cyclo­penta­dien­yl](η^4^-tetra­phenyl­cyclo­butadiene)cobalt(I)

**DOI:** 10.1107/S1600536811016928

**Published:** 2011-05-14

**Authors:** Donagh Courtney, Anthony R. Manning, C. John McAdam, Jim Simpson

**Affiliations:** a School of Chemistry and Chemical Biology, University College Dublin, Belfield, Dublin 4, Ireland; b Department of Chemistry, University of Otago, PO Box 56, Dunedin, New Zealand

## Abstract

In the title compound, [Co(C_13_H_9_)(C_28_H_20_)], the Co atom is sandwiched between cyclo­penta­dienyl and cyclo­butadienyl rings that are inclined at a dihedral angle of 2.6 (3)°. The four phenyl rings are tilted with respect to the cyclo­butadienyl plane so that the C_4_Ph_4_ unit constitutes a four-bladed propeller. The phenyl ring of the phenyl-alkyne substituent is inclined to the cyclo­penta­dienyl ring at an angle of 34.92 (18)°. The crystal structure is stabilized solely by C—H⋯π inter­actions which generate a three-dimensional network.

## Related literature

For the synthesis, see: Stephens & Castro (1963 ▶). For related structures, see: Classen *et al.* (2002 ▶); Cuffe *et al.* (2005 ▶); Kjaergaard *et al.* (2008 ▶); Zora *et al.* (2006 ▶). For recent applications of [Co(η^4^-C_4_Ph_4_)(η^5^-C_5_H_4_ *R*)] compounds, see: O’Donohue *et al.* (2011*a* ▶,*b* ▶); Nguyen *et al.* (2008 ▶).
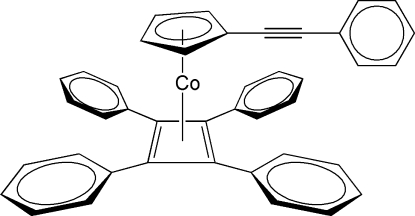


## Experimental

### 

#### Crystal data

[Co(C_13_H_9_)(C_28_H_20_)]
*M* *_r_* = 580.57
Monoclinic, 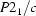

*a* = 11.2685 (5) Å
*b* = 14.9167 (7) Å
*c* = 16.8122 (8) Å
β = 97.937 (3)°
*V* = 2798.9 (2) Å^3^
*Z* = 4
Mo *K*α radiation
μ = 0.64 mm^−1^
*T* = 91 K
0.46 × 0.34 × 0.13 mm

#### Data collection

Bruker APEXII CCD area-detector diffractometer
Absorption correction: multi-scan (*SADABS*; Bruker, 2006 ▶) *T*_min_ = 0.829, *T*_max_ = 1.000
25140 measured reflections
3651 independent reflections
3130 reflections with *I* > 2σ(*I*)
*R*_int_ = 0.050
θ_max_ = 22.6°

#### Refinement

*R*[*F* ^2^ > 2σ(*F* ^2^)] = 0.045
*wR*(*F* ^2^) = 0.130
*S* = 1.06
3651 reflections
379 parameters
H-atom parameters constrained
Δρ_max_ = 1.05 e Å^−3^
Δρ_min_ = −0.48 e Å^−3^

### 

Data collection: *APEX2* (Bruker, 2006 ▶); cell refinement: *APEX2* and *SAINT* (Bruker, 2006 ▶); data reduction: *SAINT*; program(s) used to solve structure: *SHELXS97* (Sheldrick, 2008 ▶) and *TITAN* (Hunter & Simpson, 1999 ▶); program(s) used to refine structure: *SHELXL97* (Sheldrick, 2008 ▶) and *TITAN*; molecular graphics: *ORTEP-3* (Farrugia, 1997 ▶) and *Mercury* (Macrae *et al.*, 2008 ▶); software used to prepare material for publication: *SHELXL97*, *enCIFer* (Allen *et al.*, 2004 ▶), *PLATON* (Spek, 2009 ▶) and *publCIF* (Westrip, 2010 ▶).

## Supplementary Material

Crystal structure: contains datablocks global, I. DOI: 10.1107/S1600536811016928/cv5089sup1.cif

Structure factors: contains datablocks I. DOI: 10.1107/S1600536811016928/cv5089Isup2.hkl

Additional supplementary materials:  crystallographic information; 3D view; checkCIF report

## Figures and Tables

**Table 1:** Hydrogen-bond geometry (Å, °) *Cg*1 and *Cg*2 are the centroids of the C11–C16 and C17–C22 phenyl rings, respectively.

*D*—H⋯*A*	*D*—H	H⋯*A*	*D*⋯*A*	*D*—H⋯*A*
C32—H32⋯*Cg*1^i^	0.95	2.66	3.460 (4)	142
C14—H14⋯*Cg*2^ii^	0.95	2.86	3.674 (4)	144
C28—H28⋯*Cg*2^iii^	0.95	2.93	3.578 (4)	127

Symmetry codes: (i) 
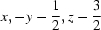
; (ii) 
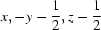
; (iii) 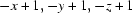.

**Fig. 1.**
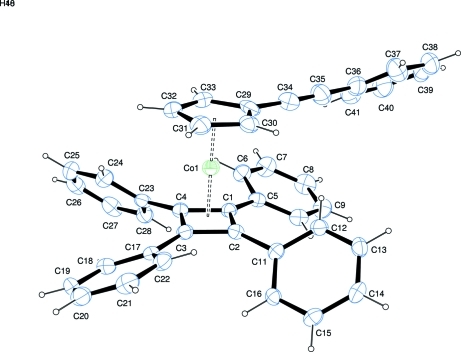
The structure of I showing the atom numbering with ellipsoids drawn at the 50% probability level.

**Fig. 2.**
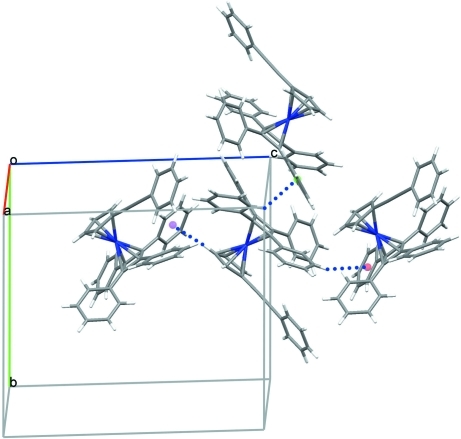
C–H···π interactions (dotted lines) in the structure of I.

**Fig. 3.**
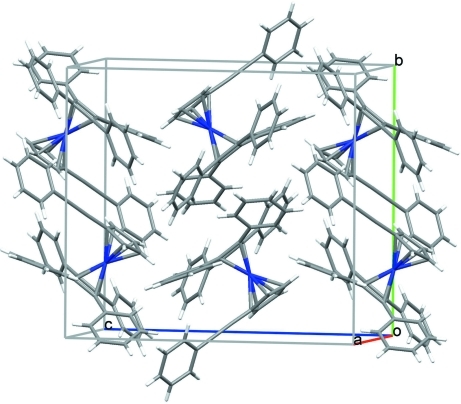
Crystal packing for I viewed down the a axis.

## supplementary crystallographic information

### Comment

Recent interest in [Co(η^4^-C_4_Ph_4_)(η^5^-C_5_H_4_R] type compounds has focused on their applications in asymmetric synthesis and catalysis, and as components of molecular machines (Nguyen *et al.*, 2008; O'Donohue *et al.*, 2011*a*). Despite this attention, comparison with its isoelectronic ferrocene cousin reveals relatively few crystallographically characterized examples. Alkyne derivatives in particular are limited to five structures (Classen *et al.*, 2002). We present here data for the archetypal derivative [Co(η^4^-C_4_Ph_4_)(η^5^-C_5_H_4_—C≡ C-Aryl)], Aryl = phenyl.

Bond lengths and angles within the [Co(η^4^-C_4_Ph_4_)(η^5^-C_5_H_4_–} fragment are close to those reported in other examples (Classen *et al.*, 2002; Kjaergaard *et al.*, 2008; O'Donohue *et al.*, 2011*a*,*b*). The distances between the cyclopentadienyl (Cp) and cyclobutadienyl (Cb) rings and the cobalt atom are 1.6759 (17) Å and 1.6990 (17) Å respectively and the angle between these planes is 2.6 (3)°. The four phenyl rings are tilted in an alternating 20.1/41.9/25.0/46.0° pattern with respect to the Cb plane so that the C_4_Ph_4_ unit constitutes a four-bladed propeller. The C≡C length of 1.207 (6) Å is similar to those observed for [Co(η^4^-C_4_Ph_4_)(η^5^-C_5_H_4_–] butadiynes (Classen *et al.*, 2002) but is slightly longer than its ferrocenyl analogue Fc—C≡ C-Ph, 1.192 (3) (Zora *et al.*, 2006) and other simple ferrocenyl ethynylaryl compounds (Cuffe *et al.*, 2005). The alkyne phenyl group is tilted 34.92 (18)° with respect to the Cp plane in marked contrast to the angle of 89.06 (13)° observed for Fc—C≡C-phenyl (Zora *et al.*, 2006).

In the crystal structure C–H···π interactions (Table 1) involving contacts from the C29···C33 Cp ring to *Cg*1, the centroid of the C11···C16 ring, and the C11···C16 phenyl ring to *Cg*2, the centroid of the C17···C22 ring, link the molecules into chains along the *c* axis (Fig. 2). An additional C28–H28···*Cg*2 contact generates a three-dimensional network structure (Fig. 3).

### Experimental

The title compound was prepared by the Castro-Stephens coupling (Stephens & Castro, 1963) of [Co(η^4^-C_4_Ph_4_)(η^5^-C_5_H_4_–C≡C–H)] and an equimolar amount of iodobenzene by stirring with 2.5 mol% CuI in N_2_ degassed triethylamine for 16 hrs. Solvent was removed under vacuum and the residue chromatographed on silica with a CH_2_Cl_2_ eluent. X-ray quality crystals were grown from CH_2_Cl_2_ layered with hexane.

### Refinement

All H-atoms were geometrically positioned, and refined using a riding model with d(C—H) = 0.95 Å, *U*_iso_=1.2*U*_eq_ (C).

### Crystal data

**Table:**

[Co(C_13_H_9_)(C_28_H_20_)]	*F*(000) = 1208
*M**_r_* = 580.57	*D*_x_ = 1.378 Mg m^−^^3^
Monoclinic, *P*2_1_/*c*	Mo *K*α radiation, λ = 0.71073 Å
Hall symbol: -P 2ybc	Cell parameters from 5542 reflections
*a* = 11.2685 (5) Å	θ = 2.3–22.4°
*b* = 14.9167 (7) Å	µ = 0.64 mm^−^^1^
*c* = 16.8122 (8) Å	*T* = 91 K
β = 97.937 (3)°	Irregular fragment, orange
*V* = 2798.9 (2) Å^3^	0.46 × 0.34 × 0.13 mm
*Z* = 4	

### Data collection

**Table:**

Bruker APEXII CCD area-detector diffractometer	3651 independent reflections
Radiation source: fine-focus sealed tube	3130 reflections with *I* > 2σ(*I*)
graphite	*R*_int_ = 0.050
ω scans	θ_max_ = 22.6°, θ_min_ = 1.8°
Absorption correction: multi-scan (*SADABS*; Bruker, 2006)	*h* = −12→12
*T*_min_ = 0.829, *T*_max_ = 1.000	*k* = −16→16
25140 measured reflections	*l* = −16→18

### Refinement

**Table:**

Refinement on *F*^2^	Primary atom site location: structure-invariant direct methods
Least-squares matrix: full	Secondary atom site location: difference Fourier map
*R*[*F*^2^ > 2σ(*F*^2^)] = 0.045	Hydrogen site location: inferred from neighbouring sites
*wR*(*F*^2^) = 0.130	H-atom parameters constrained
*S* = 1.06	*w* = 1/[σ^2^(*F*_o_^2^) + (0.0693*P*)^2^ + 4.4297*P*] where *P* = (*F*_o_^2^ + 2*F*_c_^2^)/3
3651 reflections	(Δ/σ)_max_ < 0.001
379 parameters	Δρ_max_ = 1.05 e Å^−^^3^
0 restraints	Δρ_min_ = −0.48 e Å^−^^3^

### Special details

**Table:**

Experimental. IR ν/cm^-1^ν(C≡C) 2214 (ATR). ^1^H NMR (CDCl_3_): δ 7.5 (8*H*, m, Cb-*o*-Ph), 7.42 (2*H*, m, C_2_-*m*-Ph), 7.27 (1*H*, m, C_2_-*p*-Ph), 7.2 (12*H*, m, Cb-*m/p*-Ph), 7.14 (2*H*, m, C_2_-*o*-Ph), 4.82 (2*H*, t, *α*-Cp), 4.65 (2*H*, t, *β*-Cp). ^13^C NMR (CDCl_3_): δ 135.6 (Cb-*ipso*-Ph), 131.4 (C_2_-*o*-Ph), 128.9 (C_2_-*m*-Ph & Cb-*o*-Ph), 128.1 (Cb-*m*-Ph), 127.6 (C_2_-*p*-Ph), 126.4 (Cb-*p*-Ph), 123.7 (C_2_-*ipso*-Ph), 87.8 (*C*≡CPh), 85.7 (*α*-Cp), 85.2 (C≡*C*Ph), 84.9 (*β*-Cp), 79.2 (*ipso*-Cp), 76.2 (C_4_Ph_4_). UV-Vis, λ_max_/nm (ε/dm^3^mol^-1^cm^-1^) (CH_2_Cl_2_): 269 (44 000), 300 (sh, 30 000), 330 (sh, 24 000) 385 (sh, 4800).
Geometry. All e.s.d.'s (except the e.s.d. in the dihedral angle between two l.s. planes) are estimated using the full covariance matrix. The cell e.s.d.'s are taken into account individually in the estimation of e.s.d.'s in distances, angles and torsion angles; correlations between e.s.d.'s in cell parameters are only used when they are defined by crystal symmetry. An approximate (isotropic) treatment of cell e.s.d.'s is used for estimating e.s.d.'s involving l.s. planes.
Refinement. Refinement of *F*^2^ against ALL reflections. The weighted *R*-factor *wR* and goodness of fit *S* are based on *F*^2^, conventional *R*-factors *R* are based on *F*, with *F* set to zero for negative *F*^2^. The threshold expression of *F*^2^ > σ(*F*^2^) is used only for calculating *R*-factors(gt) *etc*. and is not relevant to the choice of reflections for refinement. *R*-factors based on *F*^2^ are statistically about twice as large as those based on *F*, and *R*- factors based on ALL data will be even larger.

### Fractional atomic coordinates and isotropic or equivalent isotropic displacement parameters (Å^2^)

**Table:**

	*x*	*y*	*z*	*U*_iso_*/*U*_eq_	
C1	0.6521 (3)	0.2050 (2)	1.0157 (2)	0.0227 (8)	
C2	0.5229 (3)	0.2125 (2)	1.0222 (2)	0.0230 (8)	
C3	0.4966 (3)	0.1499 (2)	0.9545 (2)	0.0237 (8)	
C4	0.6247 (3)	0.1436 (2)	0.9479 (2)	0.0236 (8)	
Co1	0.56188 (4)	0.26618 (3)	0.92077 (3)	0.0238 (2)	
C5	0.7628 (3)	0.2358 (2)	1.0628 (2)	0.0255 (9)	
C6	0.8710 (3)	0.2326 (2)	1.0314 (2)	0.0300 (9)	
H6	0.8719	0.2138	0.9775	0.036*	
C7	0.9761 (4)	0.2564 (3)	1.0777 (3)	0.0369 (10)	
H7	1.0495	0.2522	1.0561	0.044*	
C8	0.9763 (4)	0.2865 (3)	1.1556 (3)	0.0394 (10)	
H8	1.0496	0.3022	1.1875	0.047*	
C9	0.8702 (3)	0.2934 (3)	1.1867 (2)	0.0362 (10)	
H9	0.8696	0.3164	1.2393	0.043*	
C10	0.7642 (3)	0.2669 (2)	1.1414 (2)	0.0300 (9)	
H10	0.6915	0.2700	1.1639	0.036*	
C11	0.4524 (3)	0.2540 (2)	1.0794 (2)	0.0221 (8)	
C12	0.4748 (3)	0.3418 (2)	1.1069 (2)	0.0254 (8)	
H12	0.5361	0.3759	1.0876	0.030*	
C13	0.4084 (3)	0.3795 (2)	1.1618 (2)	0.0281 (9)	
H13	0.4254	0.4389	1.1805	0.034*	
C14	0.3184 (3)	0.3316 (3)	1.1894 (2)	0.0287 (9)	
H14	0.2728	0.3577	1.2269	0.034*	
C15	0.2945 (3)	0.2448 (3)	1.1621 (2)	0.0269 (9)	
H15	0.2318	0.2117	1.1807	0.032*	
C16	0.3611 (3)	0.2061 (2)	1.1083 (2)	0.0221 (8)	
H16	0.3446	0.1463	1.0907	0.027*	
C17	0.3896 (3)	0.1023 (2)	0.9165 (2)	0.0232 (8)	
C18	0.4002 (4)	0.0193 (2)	0.8804 (2)	0.0311 (9)	
H18	0.4771	−0.0069	0.8810	0.037*	
C19	0.2997 (4)	−0.0253 (3)	0.8438 (2)	0.0380 (10)	
H19	0.3085	−0.0812	0.8182	0.046*	
C20	0.1878 (4)	0.0102 (3)	0.8439 (2)	0.0387 (11)	
H20	0.1192	−0.0214	0.8193	0.046*	
C21	0.1748 (3)	0.0917 (3)	0.8796 (2)	0.0350 (10)	
H21	0.0971	0.1165	0.8798	0.042*	
C22	0.2753 (3)	0.1382 (3)	0.9157 (2)	0.0295 (9)	
H22	0.2659	0.1948	0.9399	0.035*	
C23	0.6966 (3)	0.0865 (2)	0.9016 (2)	0.0252 (8)	
C24	0.6679 (3)	0.0760 (3)	0.8189 (2)	0.0321 (9)	
H24	0.6005	0.1066	0.7913	0.038*	
C25	0.7359 (4)	0.0216 (3)	0.7764 (3)	0.0392 (10)	
H25	0.7150	0.0150	0.7200	0.047*	
C26	0.8343 (4)	−0.0232 (3)	0.8155 (3)	0.0421 (11)	
H26	0.8820	−0.0597	0.7862	0.050*	
C27	0.8628 (4)	−0.0144 (3)	0.8978 (3)	0.0387 (10)	
H27	0.9296	−0.0458	0.9252	0.046*	
C28	0.7945 (3)	0.0398 (2)	0.9404 (2)	0.0301 (9)	
H28	0.8147	0.0452	0.9969	0.036*	
C29	0.6216 (4)	0.3951 (2)	0.9078 (2)	0.0335 (10)	
C30	0.4945 (3)	0.3952 (2)	0.9017 (2)	0.0298 (9)	
H30	0.4484	0.4271	0.9356	0.036*	
C31	0.4490 (3)	0.3399 (3)	0.8368 (2)	0.0323 (9)	
H31	0.3667	0.3281	0.8194	0.039*	
C32	0.5459 (3)	0.3053 (3)	0.8023 (2)	0.0298 (9)	
H32	0.5399	0.2662	0.7573	0.036*	
C33	0.6523 (3)	0.3376 (2)	0.8449 (2)	0.0303 (9)	
H33	0.7310	0.3241	0.8344	0.036*	
C34	0.7017 (4)	0.4436 (3)	0.9644 (3)	0.0388 (10)	
C35	0.7710 (4)	0.4866 (3)	1.0105 (3)	0.0410 (11)	
C36	0.8551 (4)	0.5366 (3)	1.0640 (3)	0.0411 (11)	
C37	0.8290 (4)	0.6205 (3)	1.0890 (3)	0.0480 (12)	
H37	0.7542	0.6475	1.0698	0.058*	
C38	0.9135 (4)	0.6667 (3)	1.1432 (3)	0.0476 (12)	
H38	0.8948	0.7245	1.1617	0.057*	
C39	1.0170 (4)	0.6311 (3)	1.1682 (2)	0.0413 (11)	
H39	1.0727	0.6637	1.2048	0.050*	
C40	1.0488 (4)	0.5471 (3)	1.1432 (3)	0.0474 (12)	
H40	1.1256	0.5227	1.1615	0.057*	
C41	0.9665 (4)	0.4998 (3)	1.0913 (3)	0.0438 (11)	
H41	0.9861	0.4417	1.0740	0.053*	

### Atomic displacement parameters (Å^2^)

**Table:**

	*U*^11^	*U*^22^	*U*^33^	*U*^12^	*U*^13^	*U*^23^
C1	0.026 (2)	0.0215 (19)	0.0215 (19)	0.0007 (15)	0.0054 (15)	0.0031 (15)
C2	0.0230 (19)	0.0213 (19)	0.025 (2)	−0.0014 (15)	0.0043 (16)	0.0013 (16)
C3	0.027 (2)	0.0217 (19)	0.0235 (19)	0.0017 (16)	0.0083 (16)	0.0021 (16)
C4	0.028 (2)	0.0210 (19)	0.0219 (19)	0.0008 (16)	0.0028 (15)	0.0031 (16)
Co1	0.0257 (3)	0.0224 (3)	0.0237 (3)	0.0001 (2)	0.0054 (2)	0.0023 (2)
C5	0.025 (2)	0.023 (2)	0.029 (2)	0.0011 (16)	0.0044 (17)	0.0046 (16)
C6	0.025 (2)	0.035 (2)	0.031 (2)	0.0000 (17)	0.0060 (18)	0.0002 (17)
C7	0.022 (2)	0.046 (3)	0.042 (3)	0.0044 (18)	0.0057 (19)	−0.001 (2)
C8	0.024 (2)	0.047 (3)	0.044 (3)	−0.0002 (19)	−0.0071 (19)	0.004 (2)
C9	0.035 (2)	0.040 (2)	0.032 (2)	0.0029 (19)	−0.0012 (19)	0.0007 (19)
C10	0.026 (2)	0.030 (2)	0.033 (2)	0.0017 (17)	0.0046 (18)	0.0046 (17)
C11	0.0200 (19)	0.027 (2)	0.018 (2)	0.0014 (15)	−0.0005 (15)	0.0005 (15)
C12	0.026 (2)	0.026 (2)	0.023 (2)	−0.0035 (16)	0.0019 (16)	0.0022 (16)
C13	0.035 (2)	0.024 (2)	0.024 (2)	0.0007 (17)	−0.0012 (17)	−0.0035 (16)
C14	0.029 (2)	0.036 (2)	0.021 (2)	0.0077 (18)	0.0047 (16)	−0.0025 (17)
C15	0.022 (2)	0.037 (2)	0.023 (2)	−0.0005 (16)	0.0046 (16)	0.0013 (17)
C16	0.0212 (19)	0.0227 (19)	0.0213 (19)	−0.0012 (15)	−0.0008 (15)	−0.0011 (15)
C17	0.030 (2)	0.024 (2)	0.0172 (18)	−0.0031 (16)	0.0054 (15)	0.0032 (16)
C18	0.037 (2)	0.028 (2)	0.030 (2)	−0.0048 (18)	0.0114 (18)	0.0021 (18)
C19	0.056 (3)	0.029 (2)	0.029 (2)	−0.019 (2)	0.008 (2)	−0.0058 (18)
C20	0.040 (3)	0.051 (3)	0.025 (2)	−0.023 (2)	0.0027 (18)	−0.0011 (19)
C21	0.024 (2)	0.051 (3)	0.028 (2)	−0.0068 (19)	−0.0016 (17)	0.004 (2)
C22	0.034 (2)	0.032 (2)	0.022 (2)	0.0005 (18)	0.0035 (16)	0.0023 (17)
C23	0.026 (2)	0.0194 (19)	0.032 (2)	−0.0026 (16)	0.0102 (17)	0.0023 (16)
C24	0.036 (2)	0.033 (2)	0.028 (2)	−0.0001 (18)	0.0074 (18)	−0.0001 (18)
C25	0.053 (3)	0.036 (2)	0.032 (2)	−0.008 (2)	0.017 (2)	−0.0084 (19)
C26	0.041 (3)	0.030 (2)	0.062 (3)	−0.009 (2)	0.033 (2)	−0.013 (2)
C27	0.028 (2)	0.028 (2)	0.061 (3)	−0.0002 (18)	0.012 (2)	−0.003 (2)
C28	0.032 (2)	0.027 (2)	0.032 (2)	−0.0004 (17)	0.0075 (18)	−0.0010 (17)
C29	0.043 (2)	0.021 (2)	0.033 (2)	−0.0109 (18)	−0.0080 (19)	0.0093 (18)
C30	0.033 (2)	0.023 (2)	0.033 (2)	0.0036 (17)	0.0048 (17)	0.0052 (17)
C31	0.036 (2)	0.030 (2)	0.029 (2)	0.0023 (18)	−0.0026 (18)	0.0074 (17)
C32	0.039 (2)	0.027 (2)	0.023 (2)	0.0005 (18)	0.0045 (18)	0.0047 (17)
C33	0.033 (2)	0.032 (2)	0.027 (2)	−0.0016 (18)	0.0084 (17)	0.0062 (17)
C34	0.046 (3)	0.031 (2)	0.040 (3)	−0.001 (2)	0.007 (2)	0.005 (2)
C35	0.043 (3)	0.036 (2)	0.043 (3)	−0.006 (2)	0.003 (2)	−0.002 (2)
C36	0.045 (3)	0.043 (3)	0.037 (2)	−0.012 (2)	0.013 (2)	−0.005 (2)
C37	0.046 (3)	0.041 (3)	0.058 (3)	−0.004 (2)	0.010 (2)	−0.007 (2)
C38	0.057 (3)	0.040 (3)	0.046 (3)	−0.017 (2)	0.011 (2)	−0.012 (2)
C39	0.045 (3)	0.045 (3)	0.036 (2)	−0.018 (2)	0.014 (2)	−0.012 (2)
C40	0.045 (3)	0.055 (3)	0.040 (3)	−0.008 (2)	0.001 (2)	−0.001 (2)
C41	0.051 (3)	0.042 (3)	0.037 (3)	−0.003 (2)	0.002 (2)	0.003 (2)

### Geometric parameters (Å, °)

**Table:**

C1—C5	1.456 (5)	C18—H18	0.9500
C1—C4	1.462 (5)	C19—C20	1.368 (6)
C1—C2	1.478 (5)	C19—H19	0.9500
C1—Co1	1.991 (3)	C20—C21	1.373 (6)
C2—C11	1.467 (5)	C20—H20	0.9500
C2—C3	1.470 (5)	C21—C22	1.393 (5)
C2—Co1	1.987 (3)	C21—H21	0.9500
C3—C4	1.466 (5)	C22—H22	0.9500
C3—C17	1.468 (5)	C23—C28	1.389 (5)
C3—Co1	1.997 (3)	C23—C24	1.392 (5)
C4—C23	1.471 (5)	C24—C25	1.382 (6)
C4—Co1	1.991 (3)	C24—H24	0.9500
Co1—C33	2.039 (4)	C25—C26	1.382 (6)
Co1—C32	2.059 (4)	C25—H25	0.9500
Co1—C29	2.059 (4)	C26—C27	1.382 (6)
Co1—C30	2.078 (4)	C26—H26	0.9500
Co1—C31	2.079 (4)	C27—C28	1.383 (6)
C5—C6	1.395 (5)	C27—H27	0.9500
C5—C10	1.398 (5)	C28—H28	0.9500
C6—C7	1.371 (6)	C29—C34	1.416 (6)
C6—H6	0.9500	C29—C30	1.422 (6)
C7—C8	1.384 (6)	C29—C33	1.439 (6)
C7—H7	0.9500	C30—C31	1.407 (5)
C8—C9	1.374 (6)	C30—H30	0.9500
C8—H8	0.9500	C31—C32	1.404 (5)
C9—C10	1.382 (5)	C31—H31	0.9500
C9—H9	0.9500	C32—C33	1.396 (5)
C10—H10	0.9500	C32—H32	0.9500
C11—C16	1.394 (5)	C33—H33	0.9500
C11—C12	1.400 (5)	C34—C35	1.207 (6)
C12—C13	1.386 (5)	C35—C36	1.424 (6)
C12—H12	0.9500	C36—C37	1.366 (6)
C13—C14	1.372 (5)	C36—C41	1.388 (6)
C13—H13	0.9500	C37—C38	1.404 (6)
C14—C15	1.388 (5)	C37—H37	0.9500
C14—H14	0.9500	C38—C39	1.297 (6)
C15—C16	1.380 (5)	C38—H38	0.9500
C15—H15	0.9500	C39—C40	1.383 (6)
C16—H16	0.9500	C39—H39	0.9500
C17—C18	1.390 (5)	C40—C41	1.377 (6)
C17—C22	1.393 (5)	C40—H40	0.9500
C18—C19	1.382 (6)	C41—H41	0.9500
			
C5—C1—C4	133.9 (3)	C16—C15—H15	119.7
C5—C1—C2	135.6 (3)	C14—C15—H15	119.7
C4—C1—C2	90.0 (3)	C15—C16—C11	120.5 (3)
C5—C1—Co1	126.6 (2)	C15—C16—H16	119.7
C4—C1—Co1	68.44 (19)	C11—C16—H16	119.7
C2—C1—Co1	68.01 (19)	C18—C17—C22	118.2 (3)
C11—C2—C3	134.9 (3)	C18—C17—C3	120.4 (3)
C11—C2—C1	135.1 (3)	C22—C17—C3	121.4 (3)
C3—C2—C1	89.5 (3)	C19—C18—C17	120.6 (4)
C11—C2—Co1	126.7 (2)	C19—C18—H18	119.7
C3—C2—Co1	68.71 (19)	C17—C18—H18	119.7
C1—C2—Co1	68.35 (19)	C20—C19—C18	120.8 (4)
C4—C3—C17	134.0 (3)	C20—C19—H19	119.6
C4—C3—C2	90.2 (3)	C18—C19—H19	119.6
C17—C3—C2	135.3 (3)	C19—C20—C21	119.8 (4)
C4—C3—Co1	68.21 (19)	C19—C20—H20	120.1
C17—C3—Co1	127.5 (2)	C21—C20—H20	120.1
C2—C3—Co1	67.98 (19)	C20—C21—C22	120.1 (4)
C1—C4—C3	90.3 (3)	C20—C21—H21	120.0
C1—C4—C23	134.7 (3)	C22—C21—H21	120.0
C3—C4—C23	134.2 (3)	C17—C22—C21	120.5 (4)
C1—C4—Co1	68.49 (19)	C17—C22—H22	119.7
C3—C4—Co1	68.65 (19)	C21—C22—H22	119.7
C23—C4—Co1	127.9 (2)	C28—C23—C24	118.2 (3)
C2—Co1—C4	63.05 (14)	C28—C23—C4	120.1 (3)
C2—Co1—C1	43.63 (14)	C24—C23—C4	121.7 (3)
C4—Co1—C1	43.07 (14)	C25—C24—C23	121.0 (4)
C2—Co1—C3	43.31 (14)	C25—C24—H24	119.5
C4—Co1—C3	43.14 (14)	C23—C24—H24	119.5
C1—Co1—C3	62.71 (14)	C24—C25—C26	120.2 (4)
C2—Co1—C33	159.37 (15)	C24—C25—H25	119.9
C4—Co1—C33	115.52 (15)	C26—C25—H25	119.9
C1—Co1—C33	119.79 (15)	C25—C26—C27	119.4 (4)
C3—Co1—C33	150.13 (15)	C25—C26—H26	120.3
C2—Co1—C32	160.72 (15)	C27—C26—H26	120.3
C4—Co1—C32	117.56 (15)	C26—C27—C28	120.3 (4)
C1—Co1—C32	150.69 (15)	C26—C27—H27	119.9
C3—Co1—C32	122.29 (15)	C28—C27—H27	119.9
C33—Co1—C32	39.82 (15)	C27—C28—C23	120.9 (4)
C2—Co1—C29	125.57 (15)	C27—C28—H28	119.6
C4—Co1—C29	140.44 (15)	C23—C28—H28	119.6
C1—Co1—C29	112.01 (15)	C34—C29—C30	126.1 (4)
C3—Co1—C29	168.57 (15)	C34—C29—C33	127.0 (4)
C33—Co1—C29	41.11 (16)	C30—C29—C33	106.9 (3)
C32—Co1—C29	67.61 (15)	C34—C29—Co1	126.3 (3)
C2—Co1—C30	112.71 (15)	C30—C29—Co1	70.6 (2)
C4—Co1—C30	175.54 (15)	C33—C29—Co1	68.7 (2)
C1—Co1—C30	133.08 (15)	C31—C30—C29	108.1 (3)
C3—Co1—C30	135.03 (15)	C31—C30—Co1	70.3 (2)
C33—Co1—C30	67.86 (15)	C29—C30—Co1	69.2 (2)
C32—Co1—C30	66.89 (15)	C31—C30—H30	126.0
C29—Co1—C30	40.20 (15)	C29—C30—H30	126.0
C2—Co1—C31	127.49 (15)	Co1—C30—H30	126.1
C4—Co1—C31	143.75 (15)	C32—C31—C30	108.4 (3)
C1—Co1—C31	169.44 (15)	C32—C31—Co1	69.4 (2)
C3—Co1—C31	115.96 (15)	C30—C31—Co1	70.2 (2)
C33—Co1—C31	67.09 (15)	C32—C31—H31	125.8
C32—Co1—C31	39.65 (15)	C30—C31—H31	125.8
C29—Co1—C31	67.17 (15)	Co1—C31—H31	126.2
C30—Co1—C31	39.56 (15)	C33—C32—C31	108.8 (3)
C6—C5—C10	118.2 (3)	C33—C32—Co1	69.3 (2)
C6—C5—C1	120.7 (3)	C31—C32—Co1	70.9 (2)
C10—C5—C1	121.0 (3)	C33—C32—H32	125.6
C7—C6—C5	120.5 (4)	C31—C32—H32	125.6
C7—C6—H6	119.8	Co1—C32—H32	125.7
C5—C6—H6	119.8	C32—C33—C29	107.9 (3)
C6—C7—C8	120.7 (4)	C32—C33—Co1	70.9 (2)
C6—C7—H7	119.7	C29—C33—Co1	70.2 (2)
C8—C7—H7	119.7	C32—C33—H33	126.1
C9—C8—C7	119.8 (4)	C29—C33—H33	126.1
C9—C8—H8	120.1	Co1—C33—H33	124.5
C7—C8—H8	120.1	C35—C34—C29	177.7 (4)
C8—C9—C10	120.0 (4)	C34—C35—C36	178.6 (5)
C8—C9—H9	120.0	C37—C36—C41	118.9 (4)
C10—C9—H9	120.0	C37—C36—C35	121.4 (4)
C9—C10—C5	120.8 (4)	C41—C36—C35	119.7 (4)
C9—C10—H10	119.6	C36—C37—C38	119.6 (4)
C5—C10—H10	119.6	C36—C37—H37	120.2
C16—C11—C12	118.2 (3)	C38—C37—H37	120.2
C16—C11—C2	120.3 (3)	C39—C38—C37	120.5 (4)
C12—C11—C2	121.4 (3)	C39—C38—H38	119.7
C13—C12—C11	120.7 (3)	C37—C38—H38	119.7
C13—C12—H12	119.6	C38—C39—C40	122.1 (4)
C11—C12—H12	119.6	C38—C39—H39	119.0
C14—C13—C12	120.4 (3)	C40—C39—H39	119.0
C14—C13—H13	119.8	C41—C40—C39	118.4 (4)
C12—C13—H13	119.8	C41—C40—H40	120.8
C13—C14—C15	119.5 (3)	C39—C40—H40	120.8
C13—C14—H14	120.2	C40—C41—C36	120.4 (4)
C15—C14—H14	120.2	C40—C41—H41	119.8
C16—C15—C14	120.6 (3)	C36—C41—H41	119.8

### Hydrogen-bond geometry (Å, °)

**Table:**

Cg1 and Cg2 are the centroids of the C11–C16 and C17–C22 phenyl rings, respectively.

**Table:**

*D*—H···*A*	*D*—H	H···*A*	*D*···*A*	*D*—H···*A*
C32—H32···Cg1^i^	0.95	2.66	3.460 (4)	142
C14—H14···Cg2^ii^	0.95	2.86	3.674 (4)	144
C28—H28···Cg2^iii^	0.95	2.93	3.578 (4)	127

Symmetry codes: (i) *x*, −*y*−1/2, *z*−3/2; (ii) *x*, −*y*−1/2, *z*−1/2; (iii) −*x*+1, −*y*+1, −*z*+1.
